# ﻿Revision of the leafhopper genus *Smyga* (Hemiptera, Cicadellidae, Typhlocybinae)

**DOI:** 10.3897/zookeys.1198.119765

**Published:** 2024-04-23

**Authors:** Michael D. Webb, Ye Xu

**Affiliations:** 1 Department of Life Sciences (Insects), The Natural History Museum, London, SW7 5BD, UK The Natural History Museum London United Kingdom; 2 School of Agricultural Science, Jiangxi Agricultural University, Nanchang 330045, China Jiangxi Agricultural University Nanchang China

**Keywords:** Auchenorrhyncha, distribution, Homoptera, morphology, taxonomy

## Abstract

The leafhopper genus *Smyga* Dworakowska (Typhlocybinae, Empoascini) is reviewed and a new species, *S.brevipenis* Webb & Xu, **sp. nov.** from Brunei and Malaysia, is described based on specimens previously identified as “aberrant specimens” of *Smygadistincta* Dworakowska. Images of the types of *S.brevipenis* and *S.distincta* are given for the first time. A checklist and key to known species of *Smyga* are also provided.

## ﻿Introduction

**Figures 1–13.**
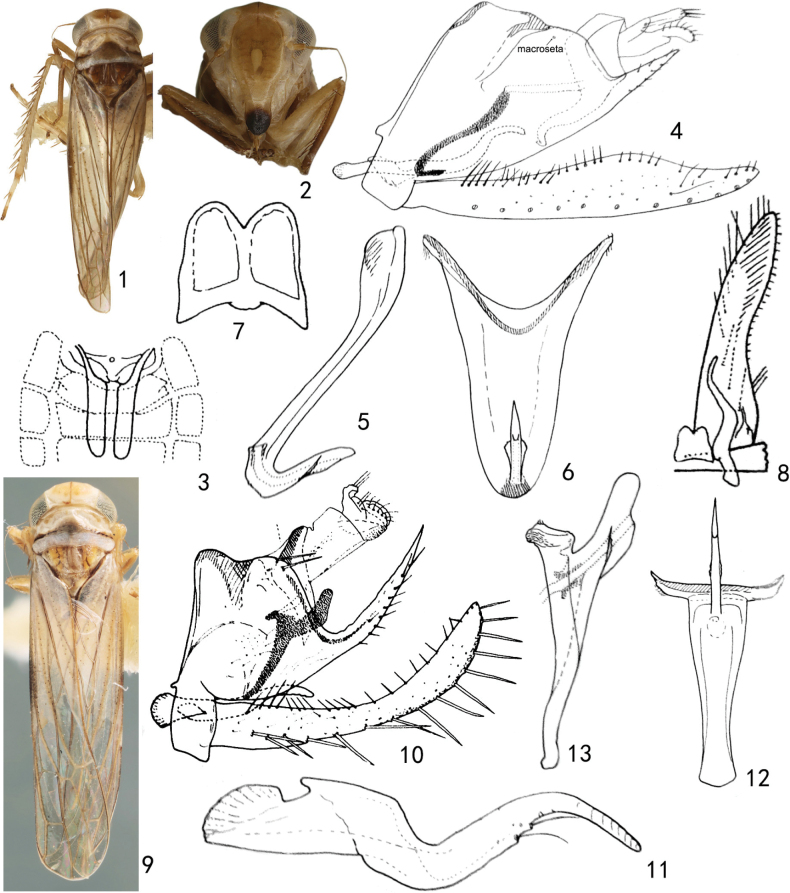
*Smyga* species **1–8***S.brevipenis***1** head and thorax, dorsal view (holotype) **2** face (holotype) **3–8** male genitalia after Dworakowska’s (1995) figures of aberrant form of *S.distincta***3** basal sternal apodemes **4** male genital capsule, left lateral view **5** aedeagus left lateral view **6** aedeagus, ventral view **7** connective **8** connective, style and subgenital plate **9–13***S.distincta***9** habitus (holotype) **10–13** male genitalia, after Dworakowska (1995)**10** genital capsule **11** style **12** aedeagus ventral view **13** aedeagus, left lateral view.

The typhlocybine leafhopper genus, *Smyga* Dworakowska, 1995, was described for five species from Brunei and Malaysia (Sarawak and Sabah) (Dworakowska 1995; Knight 2010). The genus is recognized by a brown spot on the fore margin of the head and sinuate transverse brown band on the pronotum (Figs 1, 9), a well-developed coronal suture extended onto the face (Fig. 2), and a hind wing with veins MP and CuA free distally. Although species are very similar externally, there are considerable differences in their male genitalia, particularly with respect to the aedeagus, so much so that Dworakowska (1995) tentatively regarded one species, *S.distincta*, as having an aberrant form with a remarkably short aedeagal shaft. However, the discovery of two new species from Papua New Guinea in Xu’s (2019) PhD thesis, with similar aedeagus, suggests that the specimens described and illustrated by Dworakowska as an aberrant form represent a separate species. This new species is described and illustrated below and a checklist and key to the known species of the genus are presented.

## ﻿Materials and methods

Morphological terminology used in this work follows Xu et al. (2021). Studied specimens are deposited in the insect collection of the Natural History Museum, London, UK (NHM).

## ﻿Taxonomy

### ﻿ Smyga

Taxon classificationAnimaliaHemipteraCicadellidae

Dworakowska, 1995

17410AFD-B79D-50AE-8974-05195CD017A8

Smyga Dworakowska, 1995: 151.

#### Type species.

*Smygadistincta* Dworakowska, 1995 by original designation.

#### Description.

Body relatively robust. Pale yellow; head with a brown patch anteriorly (Figs 1, 9); face with anteclypeus brown distally (Fig. 2), sometimes with more extensive brown marking (*S.divergens*); pronotum with a brown transverse narrow band at midlength, pale yellow anterior to band and silvery posterior to band (Figs 1, 9); mesonotum with yellow to brown basal triangles (Figs 1, 9).

Head including eyes broader than pronotum in dorsal view, crown short and broad, round anteriorly, length along midline shorter than one-half width between eyes; coronal suture well developed, extended onto face, and terminating at level of antennal bases (Fig. 2). Ocelli distinct, well separated from eyes (Fig. 2). Face moderately broad; lateral frontal suture well developed, curved mesad above antennal pit and meeting coronal suture at midline ventromesad of ocelli; anteclypeus slightly convex, not expanded (Fig. 2). Pronotum large with sinuate transverse depression (Figs 1, 9). Forewing narrow, rounded apically; apical cells occupying almost one-third of total length; vein R2 and RM dissociated at bases, joined by cross-vein, both arising from r cell; vein ScP+RA is not detectable; vein MCu almost parallel with vein RM apically. Hindwing with MP+CuA confluent. Front femur seta AM1 stout, situated near ventral margin; intercalary row with one large basal seta and eight or nine smaller setae near tip of femur. Hind femur with macrosetal formula 2 + 1 + 1; tibia row AV with six or seven preapical macrosetae.

Male basal abdominal sternal apodemes (2S apodemes) well developed (Fig. 3). Male pygofer elongated, strongly narrowing caudad, posterior margin acute with few rigid microsetae distally, dorsal margin with macrosetae, long fine ventrolateral setae present, ventral appendage absent (Figs 4, 10). Anal tube process elongate, extended to ventral margin of genital capsule (Figs 4, 10). Subgenital plate broadest near base (Fig. 10) or subapically (Fig. 4), all categories of setae well differentiated; basal setae encompassing mid-length of plate; marginal setae well defined; macrosetae uniserate; feeble microsetae arranged in 2–4 irregular rows apically (Fig. 4). Connective with media sclerotization, anterior margin and posterior margin deeply emarginated (Fig. 7). Style short, sinuate, with tiny teeth and microsetae preapically (Fig. 11). Aedeagus shaft short, tubular, with basal apodeme long (Figs 5, 13) or laterally compressed with basal apodeme short (Figs 6, 12); gonopore apical on ventral surface (Fig. 12).

#### Notes.

*Smyga* superficially resembles *Dapitana* Mahmood, 1967 in the features of the head and wings (coronal suture extended onto face and terminating at level of antennal bases (Fig. 2), forewing with vein R2 and RM dissociated at bases, joined by cross-vein, both arising from r cell and vein ScP+RA is not detectable). It differs from *Dapitana* in color pattern (see generic description) and in having the male pygofer with one or two dorsal macrosetae and long fine ventrolateral setae (Figs 4, 10) (pygofer without macrosetae and long fine setae in *Dapitana*). Both genera occur on both sides of Wallace’s Line, separating the Oriental from Australian regions.

*Smyga* includes five previously known species, all from Borneo, described by Dworakowska (1995). In her treatment of the type species, *S.distincta*, Dworakowska also described and illustrated some “aberrant specimens” that she excluded from the type series because she considered the small, ventrally positioned aedeagal shaft to be “not functional.” Here we recognize these specimens as a valid species: *S.brevipenis* sp. nov., as similar specimens of two new species have been seen in Xu’s (2019) PhD thesis.

As most *Smyga* species are from similar localities in Borneo (see Checklist) and as males are needed for identification, the female paratypes of two species (*S.distincta* and *S.zonata*) must be regarded as of uncertain identity (see also comments under *S.distincta* and *S.zonata*). It is also of some interest that, compared to males, the number of known females is very low.

#### Distribution.

Oriental Region (Brunei, Malaysia).

##### ﻿Checklist to species of *Smyga*

*S.brevipenis* Webb & Xu, sp. nov. (Brunei, Ulu Temburong; Sarawak, Gunong Mulu National Park)

*S.distincta* Dworakowska, 1995: 153–155, figs 168–177 (Brunei, Ulu Temburong)

*S.exhibita* Dworakowska, 1995: 155, figs 198–205 (Brunei, Ulu Temburong)

*S.niema* Dworakowska, 1995: 155–156, figs 206–212 (Sabah)

*S.ziewa* Dworakowska, 1995: 155, figs 188–192 (Brunei, Bukit Sulang)

*S.zonata* Dworakowska, 1995: 155, figs 193–197 (Sarawak, Gunung Mulu National Park)

### ﻿Key to species *Smyga* Dworakowska (males)

**Table:** 

1	Aedeagus with basal apodeme (dorsal apodeme) very long (Fig. 5)	***S.brevipenis***
–	Aedeagus with basal apodeme very short (Fig. 13)	**2**
2	Aedeagus without processes (Figs 12, 13)	***S.distincta***
–	Aedeagus with processes	**3**
3	Aedeagus with processes near base of shaft	**4**
–	Aedeagus with processes at apex of shaft	**5**
4	Aedeagus with processes near base of shaft long	***S.zonata***
–	Aedeagus with processes near base of shaft short	***S.ziewa***
5	Aedeagus with short apical processes	***S.exhibita***
–	Aedeagus with long apical processes	***S.niema***

### ﻿ Smyga brevipenis

Taxon classificationAnimaliaHemipteraCicadellidae

Webb & Xu sp. nov.

ECCEBE61-6F2E-5A95-9651-E20955DFF1C3

https://zoobank.org/23A87192-5521-448F-B080-84EC4CCEC98D

Figs 1–8

Smyga distincta Dworakowska, 1995: 153, figs 178–182, in part.

#### Material examined.

***Holotype*.** ♂, Brunei, Ulu Temburong; 300 m elev.; Feb–Mar. 1982; M.C. Day leg.; B.M. 1983-75 (NHM).

***Paratypes*.** Brunei, 8 ♂♂, same data as holotype; 10 ♂♂, same data as holotype except Ulu Temburong ridge; (NHM). Malaysia, Sarawak, Gunung Mulu National Park: 5 ♂♂, Gunung Api, 900 m elev., site 25, April; montane forest, mv and act light trap; J.D. Holloway leg.; 2 ♂♂, nr Long Melinau, 50 m elev., site 17, May; low secondary forest, mv light on river-bank; J.D. Holloway; 7 ♂♂, W. Melinau Gorge leg., 250 m elev., site 23, April; limestone forest, canopy/understory, mv light; J.D. Holloway leg.; 1 ♂, W. Melinau Gorge leg., Kerankas, 150 m elev., Mar.–Apr. 1978; J.D. Holloway leg.; 2 ♂♂, camp 2.5, 30 Apr. 1978, V.F. Eastop. All paratypes from Sarawak were collected on the RGS Mulu Expedition, B.M. 1978-206 (NHM).

#### Description.

Body length: 3.5–4.4 mm.

Color as in generic description (Fig. 1).

Male 2S apodemes not extending to end of segment V (Fig. 3). Anal-tube appendage hook-shaped at apex (Fig. 4). Male pygofer strongly emarginate dorsally in profile, and posteriorly extended into sharp point with spine-like setae; ventral appendage absent; bearing one long macroseta near dorsal margin (Fig. 4); dorsal bridge with a posterior lobe-like projection each side of midline, in dorsal view. Subgenital plate surpassing pygofer lobe, broadened medially in later view, all categories of setae represented (Fig. 4). Connective anterior margin with undeveloped median lobe, posterior margin notched medially (Fig. 7). Style sinuate (Figs 4, 8). Aedeagus without preatrium, basal apodeme well developed, plate-like; shaft with pair of lamellate lateral processes; gonopore subapical, ventrad (Figs 5, 6).

#### Etymology.

The name is derived from the Latin words *brevis* (short) and *penis*, which refers to the very short aedeagal shaft.

#### Distribution.

Brunei, Malaysia.

#### Notes.

Specimens of *S.brevipenis* were considered aberrant specimens of *S.distincta* by Dworakowska (1995) and of uncertain status. However, based on the high degree of difference between these two species and the discovery of two similar new species from Papua New Guinea noted in Xu’s (2019) PhD thesis, we describe the above specimens as a new species. All type specimens listed here (with some amendments) were identified by I. Dworakowska as the aberrant form of *S.distincta*. All are male and most have a Dworakowska genitalia dissection affixed to a card placed beneath the specimen. Three females (NHM) with identical data to the males could be the same species but, as three other species are also from these localities (see Checklist), their identity is uncertain. See also the concluding comments under the genus description. In Dworakowska’s (1995) figure of the head and thorax there are some fine lines in the basal triangles of the mesonotum. These are seen in some specimens, but it is not clear if they are markings or perhaps caused by shrinkage of internal tissue during drying.

### ﻿ Smyga distincta

Taxon classificationAnimaliaHemipteraCicadellidae

Dworakowska

C2549A40-24FB-5273-B01C-E13C8D55D3A2

Figs 9–13

Smyga distincta Dworakowska, 1995: 153–155, figs 168–177.

#### Notes.

The type series was originally stated as holotype male from Brunei, Ulu Temburong and one paratype male and two females from three different localities in Sarawak, Gunung Mulu National Park; however, the paratype male is in fact female. The identity of these three females must remain uncertain until females in the genus can be identified, particularly as other species are sympatric with *S.distincta* (see Checklist).

Aberrant specimens regarded as possibly this species in its original description are described above as a new species. Therefore, this species is known with certainty only from a single male, the holotype (NHM).

### ﻿ Smyga zonata

Taxon classificationAnimaliaHemipteraCicadellidae

Dworakowska

1FA8216A-07AB-5460-9B4F-43E1E241493D

Smyga zonata Dworakowska, 1995: 155, figs 193–197.

#### Notes.

The type series was originally stated to include a holotype male from Sarawak, Gunung Mulu National Park and one male and two female paratypes from Brunei, Ulu Temburong. The identity of these two females must remain uncertain until females in the genus can be identified, particularly as other species are sympatric with *S.zonata* (see Checklist). Therefore, this species is known with certainty only from two male specimens (NHM).

## Supplementary Material

XML Treatment for Smyga

XML Treatment for Smyga brevipenis

XML Treatment for Smyga distincta

XML Treatment for Smyga zonata

## ﻿Additional information

### Author ORCIDs

Michael D. Webb https://orcid.org/0000-0002-1312-6142

Ye Xu https://orcid.org/0000-0003-3230-7923

### Data availability

All of the data that support the findings of this study are available in the main text.
